# Supplementary material for: Are carbon emissions trading and green financial instruments synergistic? -Comprehensive quantitative research based on content analysis
Source: PLoS One. 2024 Mar 7;19(3):e0298601. doi: 10.1371/journal.pone.0298601 (PMC10919601; doi:10.1371/journal.pone.0298601)
Supplement: S1 Data — (ZIP) [file pone.0298601.s001.zip › Data/地级市Z_F工作报告.zip!/╕ú╜¿/╚2├≈╩╨/└┤╫╘╣1⁄2╓┌║┼í╛┬φ┐╦╩2╛▌═°í┐_2012.docx]

各位代表：

　　现在，我代表三明市人民政府向大会作政府工作报告，请予审议，并请市政协各位委员和其他列席人员提出意见。

　　一、过去五年工作回顾

　　2007年以来，市政府在省委、省政府和市委的正确领导下，在市人大及其常委会、市政协的监督支持下，坚持以邓小平理论和“三个代表”重要思想为指导，认真贯彻党的十七大以来的一系列重大决策部署，深入落实科学发展观，围绕福建发展和海西建设大局，持续实施“五大战役”，全力推动科学发展、跨越发展。经过全市人民的共同努力，经济保持较快增长，各项事业全面进步，市十一届人大一次会议确定的主要目标任务顺利完成。

　　——科学发展跨越发展进入新阶段。全市上下抓住机遇，乘势而上，“十一五”规划全面完成，“十二五”发展开局良好。海西三明生态工贸区列入全省10个新增长区域，《海西三明生态工贸区发展规划》获省政府批复实施，三明发展站在了更高的起点上。

　　——综合实力明显增强。五年全市地区生产总值、财政总收入实现翻番，成为全省第四个人均地区生产总值突破5000美元的设区市，2011年地区生产总值突破1200亿元，财政总收入突破100亿元。

　　——人民群众得到更多实惠。城乡居民收入增长与经济发展同步，实现年均两位数增长。在全国率先实行新型农村合作医疗补充保险,在全省率先消除城镇“零就业家庭”，城乡居民社会养老保险全覆盖,实现城乡免费义务教育，建成三明市新体育场馆等一批公用设施，民生福祉显著改善。

　　——产业集聚取得重大进展。中国重汽海西载重汽车等一批重大项目投产。八大产业规模工业总产值占全市规模工业的89.3%，1个产业产值超400亿元，4个产业超200亿元，2个产业超100亿元，海西先进制造业重要基地逐步形成。

　　——基础设施实现跨越。我市被确定为国家公路运输枢纽，高速公路通车里程居全省第一，2条快速铁路和三明沙县机场动工建设，形成基础设施持续建设、有效接续的良好态势，对外开放综合通道不断拓宽。

　　——改革开放形象持续提升。集体林权制度改革继续走在全国全省前列。海峡两岸林业博览会暨投资贸易洽谈会升格为国家级会展。中国丹霞·泰宁列入世界自然遗产名录。成功承办省第七届农民运动会。获得世界客属第25届恳亲大会举办权，有力地扩大了三明的对外影响。

　　五年来的主要工作是：

　　（一）注重结构调整，转变发展方式。扶持培育百亿十亿重点工业企业，工业对经济增长的贡献率达到60%以上。新增4个省级经济开发区，园区产业集群发展壮大。培育战略性新兴产业，生物医药及生物产业产值年均增长40%，不锈钢复合板、轻合金、镍合金、氟化工等新材料产业项目加快实施。在保护基本农田、稳定粮食播种面积和产量的同时，做强做优特色农业，农业产业化经营总额突破400亿元。深入开展加快流通产业发展试点城市工作，海鑫钢材交易市场列入全省重点扶持的十大商贸市场，三明公路港成为全省首家物流信息平台试点建设单位。编制实施旅游发展总体规划，游客接待人数、旅游总收入持续增长，“泰宁路径”成为全国第五个县域旅游发展模式。拓展消费市场，一批知名商贸企业入驻三明。建筑业、房地产业稳步发展。实施品牌战略，新增12个国家级旅游品牌、8个中国驰名商标和1个中国名牌产品。

　　加快科技进步。五年组织实施316个省级以上科技计划项目，专利申请量、授权量年均增长30%以上，高新技术产业产值年均增长1倍以上。“6·18”合同项目数、总投资额持续位居全省前列。加大人才引进力度，五年组建10个院士工作站，成立全省首个院士专家服务中心，累计引进高层次人才和紧缺急需专业本科以上毕业生1375人。

　　强化节能减排和生态保护。单位生产总值能耗降低和二氧化硫、氮氧化物、化学需氧量、氨氮减排任务预计可以完成。五年关闭96家“五小”企业，新建8座县级生活垃圾无害化处理场，实现县县有污水处理厂。市区空气质量达到国家二级标准，县级以上饮用水源水质达标率99.7%，辖区三条主要水系省控断面水质达标率99%。完成造林绿化面积321.8万亩，森林覆盖率继续保持全省前列。

　　（二）注重夯实基础，推进城乡建设。五年实施省、市重点项目496个，全社会固定资产投资累计超过3000亿元。新增高速公路317公里，改造省道445公里，完成路面改善工程263公里。开工建设快速铁路223.5公里。建成500千伏超高压变电站，新增电力装机容量43.7万千瓦，三明核电公司挂牌成立。“六千”水利工程等农业基础设施加快建设，防灾减灾体系不断健全，信息化建设稳步推进。

　　加快城乡发展。中心城市规划建设全面展开，三明市区、沙县和永安同城化工作逐步推进，市区道路、公共设施建设和城市景观、建筑立面改造成效明显，市区建成区面积从2006年的23.6平方公里增加到2011年的28.8平方公里。县域城市特色进一步显现，县域经济实力增强，城镇化率从2006年的42.3%提高到51.6%。推进小城镇改革发展，4个省级综合改革建设试点镇和15个市级中心乡镇规划建设有序推进。加快新农村建设，五年筹资56亿元，完成50个新农村办实事项目，1367个村通过省级农村家园清洁行动验收，通村公路硬化率达100%。“造福工程”搬迁6.1万人。解决农村91.4万人饮水安全问题。设立促进苏区老区发展专项资金，老区群众生产生活条件明显改善。加大扶贫开发力度，少数民族乡村和库区移民自身发展能力得到提升。

　　（三）注重改革创新，增强发展活力。推动农村土地承包经营权流转，成立全省首家县级土地承包流转服务中心和信托公司。永安市被确定为全国林业改革与发展示范区。深化部门预算编制改革，创新行政事业单位国有资产管理体制，市直行政事业单位资产实行集中管理和营运。三明农村商业银行开业，沙县被国家确定为全省唯一的农村金融改革试验区，3家企业境内外上市，组建三明城市建设投资公司等7个投资管理运作平台。实施政府机构改革，完成全市乡镇机构改革任务。落实促进非公有制经济发展政策，民间投资、非公有制企业总产值、上缴税收分别比2006年增长2.9倍、3.2倍、3.3倍。沙县人创业精神、永安创新经验和泰宁城建模式得到省上充分肯定。

　　扩大对外开放。五年累计开展区域协作项目1164个，引进内资361.3亿元。强化与中央属企业合作，在建、签约合作项目13个，完成投资28.8亿元。注重对接外企项目，可比口径实际利用外资、出口总值实现翻番。加强明台交流合作，海峡两岸（三明）现代林业合作实验区、海西三明台商投资区加快建设，清流台湾农民创业园升格为国家级园区，成功举办海峡两岸乡镇对口交流会，新批办台资企业88家，合同利用台资1.45亿美元。深化明港澳合作，加强与海外华侨及国际友城联系。海关、检验检疫、贸易促进等工作取得新成效。提前完成对口支援四川地震灾区恢复重建任务，扎实推进对口帮扶新疆工作。

　　（四）注重民生优先，改善人民生活。五年筹资60亿元，实施80个为民办实事项目。积极创建国家级创业型城市，在全省率先建立统筹城乡就业服务平台，新增城镇就业12.58万人，农村劳动力转移就业24.34万人。实现工伤、生育、医疗保险市级统筹，加强被征地农民社会保障工作，提高城乡低保标准。建设保障性住房32369套、竣工17291套，解决了约5万人住房难问题。保障市场供应，稳定蔬菜等副食品价格，2011年物价涨幅低于全省平均水平。深入推进“餐桌污染”治理，加强食品药品安全监管。团结抗击自然灾害，做好灾后恢复生产和重建家园工作，受灾群众生产生活得到妥善安置。加快教育发展，10个县（市、区）实现“双高普九”目标，在全省率先实施并全面推广农村义务教育阶段学生食宿改善工程和“周末学生班车”，组建6个行业型职教集团，高考成绩持续位居全省前列，学前教育、特殊教育、高等教育稳步发展。推进医药卫生体制改革，医疗卫生服务体系不断健全，基本药物制度全面实施，千人医疗机构床位数、卫技人员数高于全省平均水平，基本完成县乡村三级医疗机构标准化建设。完善基层公共文化设施，建成104个达标乡镇综合文化站，5个项目入选国家级非物质文化遗产名录，广播电视、新闻出版事业稳步发展。7个县（区）荣获全国科普示范县（区）称号。社会科学取得新进展。全民健身运动深入开展，竞技体育水平明显提高，获得5个世界举重冠军。持续稳定低生育水平，5个单位获得国家级计划生育优质服务先进称号。加强和创新社会管理，完善村（居）民自治，建立基层综治信访维稳“1＋N”联动工作模式，保持全国社会治安综合治理优秀地市称号，12个县（市、区）全部达到平安县（市、区）标准。强化安全生产工作，有效防范重特大安全生产事故发生。深入开展以创建全国文明城市为重点的多城同创活动，保持国家卫生城、国家园林城、全国双拥模范城等称号，第四次荣获全国创建文明城市工作先进城市称号，荣获全国无偿献血先进城市、全国残疾人工作示范城市、全国法制宣传教育先进城市等称号。

　　（五）注重转变职能，优化政府服务。认真执行人大及其常委会决定决议，依法接受法律监督和工作监督，负责任地办理人大代表意见建议。积极支持人民政协履行职能，自觉接受民主监督，认真办理政协提案。广泛听取、认真采纳各民主党派、工商联、各人民团体、无党派人士和社会各界人士的意见建议。出台重大事项决策程序规定、重大行政决策听取意见和听证制度。改进行政审批制度，市、县行政服务中心和乡镇“三农”服务中心得到加强，五年市级行政审批项目削减38%。落实“拉练”检查成果，激发比学赶超、干事创业的精气神。坚持“四个不让”服务准则，推进“作风建设年”和“五比”竞赛活动，投资环境总体满意度居海西20个城市前列。加强政府法治建设，推进市县政府依法行政。深入开展纠风工作，完善办事公开制度。加强政府层级监督和行政监察、审计监督，接受群众和社会舆论监督，从严治政进一步落实。完成第二次全国经济普查、第六次全国人口普查的各项任务。加快发展社会福利事业，理顺红十字会管理体制，成立市慈善总会。老龄、工会、青少年、妇女儿童工作不断加强，民族宗教、水文、气象、防震和地方志工作取得新进展。人民防空、民兵预备役和国防后备力量建设进一步加强。回顾过去五年，我们深切体会到，做好政府工作，必须坚持把科学发展跨越发展作为最重要、最迫切的任务，以改革为动力，以项目为抓手，创新运作机制，全力做大总量，着力提升质量，增强发展后劲。必须坚持把改善民生作为工作的出发点和落脚点，从事关群众切身利益的事情入手，办好为民实事，落实惠民政策，让人民群众共享改革发展成果。必须坚持把统筹协调作为推动工作的有效方法，在抓好经济建设的同时，加快社会事业发展，加强和创新社会管理，促进社会和谐稳定。必须坚持把依法行政作为各级政府的基本准则，注重公开、公平、公正，做到务实、廉洁、开明、高效，主动落实、兑现承诺，切实提高政府的执行力和公信力。各位代表，过去五年发展成就来之不易，这是在省委、省政府和市委的正确领导下，在历届政府打下的良好基础上，经过全市上下奋力拼搏取得的。在此，我代表市人民政府，向辛勤工作在各条战线的全市人民，向给予政府大力支持与有效监督的人大代表、政协委员、各民主党派、工商联、各人民团体、无党派人士、离退休老同志、社会各界人士和全体纳税人，向为三明发展做出积极贡献的中央和省属驻明单位、驻明部队、武警官兵、公安政法干警，向关心支持三明发展的港澳台同胞、海外侨胞、国际友人，致以衷心的感谢和崇高的敬意！我们清醒地认识到，经济社会发展还存在不少困难与问题：经济总量不大，自主创新能力不强，产业集群化程度不高，支撑发展后劲的大项目、好项目不多，经济结构不尽合理。中心城市辐射带动作用不突出，县域经济实力不强，农村基础设施薄弱，城乡区域发展不平衡。资金供求矛盾突出，重点项目、小微企业融资难度很大。节能减排、耕地占补平衡、生态环境保护压力加大。社会事业发展相对滞后，城乡居民持续增收面临不少困难，改善群众生活还有许多亟待解决的问题。影响社会和谐稳定的因素依然存在。政府职能、管理方式、思想观念还需继续转变，个别部门工作人员办事效率、服务水平还需进一步提高。对此，我们将高度重视，采取有力措施加以解决。

　　二、今后五年工作目标和主要任务

　　今后五年，是三明在更高起点上推进科学发展跨越发展的重要时期，也是提前三年实现全面建设小康社会目标的关键时期。我们要立足三明实际、遵循发展规律，坚持“三化”并举、“三群”联动，坚持速度与质量统一、富民与强市统一，坚持“一轴”与“两翼”协同发展，实现好中求快、又好又快的发展。今后五年政府工作总的要求和奋斗目标是：以邓小平理论和“三个代表”重要思想为指导，深入贯彻落实科学发展观，坚持以人为本、执政为民理念，按照省第九次党代会和市第八次党代会的部署，紧紧抓住福建发展和海西建设的大好机遇，围绕科学发展这个主题和加快转变、跨越发展这条主线，突出发展，注重运作，凝聚合力，弘扬正气，深入实施“工业强市、生态立市、科教兴市、开放活市”发展战略，进一步解放思想、扩大开放、转型发展、奋力跨越，坚持更高起点，着眼更高要求，全面推进海西三明生态工贸区建设，全力做大经济总量，提升发展质量，统筹城乡、区域和经济社会协调发展，切实保障和改善民生，加快建设开放三明幸福三明，努力开创科学发展跨越发展的新局面。到2016年，全市地区生产总值、人均地区生产总值、财政总收入实现翻番，力争城乡居民收入实现倍增。

　　（一）突出做大总量，提升发展质量。继续打好“五大战役”，全力对接“三维”项目,持续推进重点建设，力争投资增速超过全省平均水平。夯实“三农”基础。以工业化理念发展农业，靠现代科学技术提升农业，用现代经营方式拓展农业，重视粮食生产和粮食安全，大力发展设施农业、高优农业、观光农业、生态农业，到2016年农业产业化经营总额达到700亿元。加大新型农民培训力度，加快发展农民专业合作社，培育农产品知名品牌。健全农村社会保障体系，促进农民收入持续较快增长。加强新农村建设，以规划为龙头，统筹实施农村“五通”建设和“造福工程”，抓好农村环境综合整治和农村饮水安全工程，改善农村生产生活条件。做大产业规模。推进主导产业高端化、传统产业新型化、新兴产业规模化，到2016年形成汽车及机械制造千亿产业集群，建成冶金及金属制品深加工、林产品深加工、产业用纺织品以及氟、硅和有色金属新材料等4个500亿元以上产业集群。着力在稀土、生物医药及生物、化工新材料、环保装备、新能源等领域取得突破，力争战略性新兴产业增加值占地区生产总值10%以上。引导传统服务业向专业化、品牌化方向提升，推动物流业与制造业联动发展，促进旅游资源大市发展为旅游产业强市，扶持金融、会展、信息和社区服务等服务业发展，培育文化产业成为新的增长点，切实提高第三产业增加值占地区生产总值的比重。做特县域经济。依托特色资源，开发特色产品，把优势产业做出特色，把特色产业做出规模。支持东南一翼大力发展资源转化率高、产业协作性强、开放程度大的特色经济，打造沿海产业转移基地，把大县做成强县、优势转为实力。推动西北一翼形成充满活力、各具特色的县域经济发展格局，鼓励突出特色、壮大总量、提升质量、争先进位。做强优势企业。围绕壮大实体经济，扶持龙头企业，提升现有企业，对接大型企业。鼓励各种所有制企业发展壮大，重视老企业、兴办新企业。支持企业扩大产能、延伸产业链，引导企业重组并购、发行债券、上市融资。鼓励企业争创和经营品牌，提高综合竞争力。强化转型创新。围绕建设创新型城市，增强自主创新能力，支持地方、企业、高校、科研院所联合突破产业发展关键共性技术，切实发挥科技创新驱动作用，到2016年全社会科技研发投入达到全省平均水平。重视知识产权培育和保护。实施人才战略，加强人才引进和培养，完善人才政策，创新人才机制，优化人才环境，让各类人才拥有更好的创业平台和发展空间。

　　（二）突出改善民生，增强群众幸福感。确保民生投入增长高于财政经常性收入增长，确保新增财力更多用于改善民生和发展社会事业，着力解决人民群众最关心最直接最现实的利益问题。

　　围绕群众增收需求，大力开发就业岗位，重点解决大中专毕业生、农村转移劳动力、城镇就业困难人员的就业问题，城镇登记失业率控制在4%以内；提高居民收入在国民收入中的比重和劳动报酬在初次分配中的比重，提高中低收入群体收入水平，提高城乡低保标准和补助水平，努力缩小城乡、区域、行业和社会成员之间的收入差距。围绕群众社会保障需求，扩大各类社会保险覆盖面，逐步提高统筹层次和保障水平；健全城乡居民最低生活保障制度，完善社会救助体系，推进残疾人社会保障和服务体系建设；加快保障性安居工程建设，逐步解决城市中低收入家庭的住房困难。围绕群众公共服务需求，解决学前教育“入园难”问题，全面实施义务教育学校标准化建设，促进义务教育均衡发展，大力发展职业教育，提升高等教育水平，广泛开展社区教育、老年教育，构建终身教育体系；加强公共文化基础设施和服务网络建设，发展公益性文化事业，打响特色文化品牌，培育文化产业龙头企业；深化医药卫生体制改革，全面推进公立医院改革，健全基本医疗卫生制度，提高医药卫生管理水平，完善覆盖城乡居民的基本医疗卫生服务体系；加强公共体育场馆建设，持续开展全民健身运动，提高竞技体育水平。围绕群众权益需求，关爱老年人，重视老干部工作，积极发展老龄事业，切实维护妇女儿童合法权益，支持社会福利和慈善事业发展，在全社会倡导关心弱势群体、扶助困难人员的风气。

　　（三）突出城乡统筹，推动区域协调发展。完善规划、合理布局，统筹城乡建设、产业发展和生态保护，构建生态工贸区产业群、城镇群、交通网、生态链，实现工业化、城镇化、生态化互动并进。加快生态工贸区建设。坚持高标准，立足高起点，体现前瞻性，提高中心城市规划建设管理水平，推进三明市区、沙县和永安同城化进程，完善区域开发管理体制和运行机制，促进产业、人口和生产要素集聚，打造中心商务圈、城市综合体、休闲娱乐区，到2016年区内生产总值超过1200亿元，海西先进制造业的重要基地、海西中部现代服务业集聚地、海西重要的综合交通枢纽、福建中部宜居宜业中心城市、福建生态低碳发展示范区建设取得突破，成为海西经济发展的重要增长极。完善基础设施。建设三明国家公路运输枢纽，构建“一纵三横三联”高速公路网和“两纵两横”铁路网，实现三明沙县机场通航。争取动工建设三明核电项目。推进三明电网跨越提升工程。加快以水利为重点的农业基础设施建设，健全自然灾害防控体系，增强防灾减灾能力。加强信息基础设施建设，提升“数字三明”建设水平。加快城镇化进程。坚持规划先行、典型引路、设施完善、服务配套，合理安排产业聚集、村落分布、生态涵养等布局，推进县域城镇建设，形成一批工业强镇、商贸重镇、旅游旺镇和历史文化名镇，力争全市城镇化率每年提高1.5个百分点。用好用足支持和促进革命老区加快发展的各项扶持政策，实施老区村跨越发展工程。加大整村扶贫开发力度，支持少数民族乡村、偏远乡村和水库库区加快发展。加大生态保护和建设力度。构建生态安全格局，保护生态系统稳定。发展循环经济、低碳经济，加强节能减排工作。加快实施“四绿”工程，支持森林县城建设，保持森林覆盖率位居全省前列，争创国家级生态市、国家生态园林城市。加强国土资源管理，合理开发土地、矿产资源。

　　（四）突出先行先试，提高改革开放水平。把解放思想贯穿于发展的全过程，推动改革向纵深发展、开放向更高水平提升。推动重点领域和关键环节改革攻坚。稳步推进农村综合改革，持续深化集体林权制度改革，健全土地承包经营权流转市场。完善国有资产监管体制，深化财政体制改革。用好现有投资管理运作平台，加强矿产、旅游、林产等资源整合，提高资源资产资本运作水平。开展行政管理体制改革。赋予县（市、区）更多发展自主权，加快强镇扩权步伐。稳步推进事业单位分类改革，抓好教育、文化、医药卫生等社会事业领域的改革。支持金融创新发展，优化金融资源配置，完善金融服务体系。推动国有企业发展壮大，进一步鼓励和引导非公有制经济发展，激活民间资本，拓展商会功能，做大总部经济。

　　大力发展开放型经济。加快转变外贸发展方式，调整外贸出口结构，不断提高利用外资水平。依托海峡两岸现代林业合作实验区、三明台商投资区、清流台湾农民创业园等平台，以海峡两岸林业博览会暨投资贸易洽谈会为载体，扩大明台合作领域。拓展区域合作、山海协作，深化明港澳侨交流合作，鼓励出国人员回乡创业。

　　（五）突出社会建设，促进社会和谐稳定。提高社会管理科学化水平，最大限度激发社会活力，最大限度增加和谐因素、减少不和谐因素。

　　创新社会管理体制机制，健全社会管理格局。推进城乡社区建设，逐步推行社会管理“网格化”。落实重大决策、重大项目、重大事项社会风险评估机制，从源头上预防社会矛盾产生。在稳定低生育水平的同时，创新流动人口服务管理，推进户籍管理制度改革。做好新形势下群众工作，按照“路线图”依法处理信访事项，有效化解社会矛盾。深化“平安三明”创建，推进治安“大防控体系”建设，争创全国综治“长安杯”。加强社会主义核心价值体系建设，以“满意在三明”为主题，以“三明、三创、三促”为抓手，以“好在共建、贵在坚持、重在建设”为机制，争创全国文明城市和未成年人思想道德建设工作先进城市。

　　形成促进和谐的合力。加强民主法制建设，主动接受人大及其常委会的法律监督、工作监督和政协的民主监督，密切与各民主党派、工商联、无党派人士的联系。加强政府自身建设，推进依法行政，提高机关效能。支持工会、共青团、妇联等人民团体依照法律和各自章程开展工作。争创“六五”全国法制宣传教育先进城市。抓好民兵预备役、国防动员、人民防空、双拥共建工作。促进统计、科普、民族宗教、档案、地方志和红十字会等工作全面进步。

　　三、2012年主要工作

　　今年是实施“十二五”规划承上启下的重要一年，按照市委八届二次全会的部署，我们要进一步在做大总量中优化结构，在转变方式中促进跨越，在扩大开放中增创优势，在加快发展中改善民生，更高水平地推进经济社会发展，实现稳中求进，进中求好，进中求快，更好更快。今年我市经济社会发展主要预期目标是：全市地区生产总值增长12.5%；地方级财政一般预算收入增长15%；全社会固定资产投资增长21%；外贸出口增长12%，按可比口径实际利用外商直接投资增长12%；城镇登记失业率控制在3.8%以内；社会消费品零售总额增长18%，居民消费价格总水平涨幅控制在4%左右；人口自然增长率控制在7.5‰以内；城镇居民人均可支配收入增长12%，农民人均纯收入增长12%；完成省里下达的单位生产总值能耗降低和主要污染物减排年度任务。围绕上述目标，着重做好以下八个方面工作：

　　（一）着力抓好“三农”工作，提高农产品供给保障能力

　　加快发展现代农业。完善粮食生产扶持政策，确保粮食播种面积稳定在340万亩、粮食总产稳定在115万吨。实施“十百千万”工程，完成水利投资18亿元以上，推进一批水利重点骨干项目。壮大10个特色农业产业，建设10个特色农业基地，培育一批现代农业示范片区。扶持30家产值超亿元龙头企业，力争市级以上农业产业化龙头企业产值突破200亿元。重视农业科技队伍建设，组建市农业科技创新中心，推广农业“五新”成果，加强动植物疫病防控，提高农产品质量安全水平。加快农业标准化生产，推进农民专业合作社建设，建立农产品信息服务平台，加强农村“六大员”队伍建设，健全农业社会化服务体系。抓好10.6万亩农业综合开发土地治理项目，实施土地开发整理3万亩。

　　落实强农惠农富农政策。及时兑现农资综合补贴、良种补贴、粮食直补、农机具购置补贴等政策，扩大以工代赈规模，保障农民土地承包经营权流转增值收益。在挖掘农业内部增收潜力、巩固提高家庭经营收入的同时，创造条件增加农民工资性、转移性、财产性收入。提高农民职业技能和就业创业能力，拓展农村二、三产业。提高扶贫标准，实施扶贫开发“四大工程”，稳定解决扶贫对象温饱。

　　推进新农村建设。抓好4个省级试点镇和15个市级中心乡镇建设，编制村庄建设规划，强化农村住房建设管理，加强新型农村社区建设，完成“造福工程”搬迁3.7万人。开展农村家园清洁行动，推进农村饮水安全工程，加快“三旧”改造，抓好农村环境连片整治，推进高速公路、快速铁路沿线及重点路段、重要节点、重要区域的村镇改造。组织实施一批老区村跨越发展工程项目，推动革命基点村和重点老区村发展，支持有条件的县（市、区）申报苏区。支持少数民族乡村发展，做好库区移民安置和后期扶持工作。

　　（二）着力抓龙头铸链条，大力发展实体经济

　　推进工业集群化发展。抓紧实施推动工业跨越发展的实施意见，重点培育27家龙头企业，拓展延伸重点产业链，推动全市规模工业增加值实现较快增长。策划一批5亿元以上重点工业项目，实施150个亿元以上重点项目，抓好175个新增长点项目，突破一批重大项目。加快14个重点工业园区开发建设，提高投资强度，引项目、出效益。落实扶持中小微企业发展的政策措施，帮助企业开展融资、协调用工、提升管理、加强协作，推动企业持续健康发展。

　　促进服务业提速提质。加快海西三明现代物流产业开发区建设，推进重点物流和市场项目建设，抓好市区重点现代服务业项目，积极引进大型物流企业，大力发展商贸、金融、商务、信息、会展等服务业，加快发展家政、养老、社区服务等家庭服务业。扶持建筑业企业上等级，推动建筑业加快发展。加强房地产市场监管，保持房地产市场稳定发展。发挥泰宁旅游龙头作用，拓展旅游品牌效应，抓好10个重点旅游项目，实现游客接待人数、旅游总收入较快增长。

　　强化创新驱动和人才支撑。实施20个以上重点高新技术产业化项目，新增10家以上国家级和省级创新型（试点）企业，力争专利授权量增长20%以上，高新技术产业产值增长20%以上。加强科技创新平台建设，鼓励创建科技企业孵化基地，支持企业成立研发机构，提升企业创新能力。鼓励和支持企业争创品牌，力争“6·18”合同项目数、总投资额、动工率居全省前列。加强院士工作站、博士后科研工作站建设，培养科技创业创新人才，推行定期服务、短期服务、异地服务等人才使用模式，吸引在外地的明籍高层次人才为家乡服务。

　　（三）着力打好“五大战役”，增强投资与消费的拉动力

　　拓展战役领域和内涵。把农林水利、“四绿”工程、城镇美化、造福工程、饮水安全、防灾减灾、旅游等领域的项目纳入战役统筹实施。全年重点项目建设战役实施项目300个，计划投资350亿元，开工建设80个、竣工投产90个；新增长区域发展战役实施项目155个，计划投资205亿元，开工建设30个、建成投产30个；城市建设战役实施项目282个，计划投资96亿元；小城镇改革发展战役实施项目468个，计划投资71亿元；民生工程战役结合实施社会事业基本公共服务均等化行动计划，计划投资45亿元，实施民生项目65个。

　　加强“三维”项目对接。深化与中央属企业的合作，抓好8个在建、新开工和前期项目。加大民企项目的对接落实力度，筹备参加福建省珠三角民企项目对接洽谈会，确保上年签约合同项目动工率达80%以上。加强与港澳台等重点客商的联络，力争新签约合同项目利用外资3亿美元以上，合同项目批办率达50%以上。

　　强化重大基础设施建设。计划完成公路投资63亿元，加快建设建泰和厦沙、永漳高速公路三明段，动工建设湄州湾至重庆高速公路三明段；续建和新开工省道172公里，建成农村公路150公里。计划完成铁路投资21.5亿元，基本建成向莆铁路三明以北路段，力争全线开工南三龙铁路三明段，推进浦建龙梅、长永泉铁路项目前期工作。三明沙县机场计划投资6.5亿元。推进三明核电项目前期工作。计划完成电网投资11亿元以上，开工建设一批输变电工程，改造提升农村电网，推进500千伏三阳变二期扩建和永安输变电项目前期工作。

　　扩大消费需求。落实促进消费政策，发展新兴消费业态，扩大家电、汽车、家装等消费需求，培育发展大型超市、农贸市场和专业市场，新建一批社区便利店等消费网点。改善城乡消费环境，巩固提升“万村千乡市场工程”，支持发展消费信贷。治理“餐桌污染”，建设“食品放心工程”，加强药品监管，保障群众放心消费。抓好“米袋子”、“菜篮子”工程，推进副食品基地建设，加强重要商品和生活必需品的储备、供应，强化价格监测预警，保持物价基本稳定。

　　（四）着力保障和改善民生，加强和创新社会管理

　　强化就业与社会保障。落实更加积极的就业政策，新增城镇就业2.4万人，农村劳动力转移就业4万人。落实最低工资制度，健全职工工资正常增长机制和支付保障机制，提高企业职工特别是一线职工工资收入。加快保障性安居工程建设，新建保障性住房和改造棚户区9500套，确保质量和进度，完善分配、管理、退出等制度，扩大住房公积金覆盖面，增加普通商品住房供给。提高企业退休人员基本养老金，做好被征地农民社会保障工作。实行失业保险市级统筹，着力推进农民工参保。全面推动重大疾病住院费用补充补偿，开展新农合普通门诊统筹补偿，城镇居民医保和新农合政府补助标准提高到每人每年240元。提高农村低保标准，改善优抚对象、革命“五老”人员待遇，加强城乡困难家庭专项救助和临时救助，健全社会救助和保障标准与物价上涨挂钩联动机制。推进城市公交发展，优化公交线路，方便群众出行。发展社会福利、慈善事业，建设残疾人综合服务中心。做好老龄工作，推进社区居家养老服务中心建设，推行互助型农村养老服务模式，保障妇女儿童合法权益。

　　繁荣发展社会事业。实施学前教育发展三年行动计划，推进义务教育均衡发展，全面完成“双高普九”任务，开展教育强县创建工作；建设校舍安全等项目，抓好“校车安全工程”，实施农村义务教育阶段寄宿生营养改善工程；推动高中教育发展，创新发展职业教育，加快市职教园区、示范性综合实践基地和徐碧学校建设，力争创建3所国家级中等职业教育改革示范学校、1所国家级重点中等职业学校；推进高校重点学科建设，提升高校教学质量和科研水平；重视发展老年教育、特殊教育、民族教育、社区教育，支持民办教育发展。加快医药卫生体制改革，开展基层医疗卫生机构综合改革，整合医疗卫生资源，实施公立医院改革，建成台江医院并投入使用，启动三明儿童医院建设；加强全科医生、乡村医生的培训和配备，推进社区康复医疗试点，实现每个行政村都有1所达标卫生室；加强重大疾病防控和卫生监督，扶持中医药事业发展，加快发展妇幼保健事业。稳定低生育水平，综合治理出生人口性别比偏高问题，积极开展诚信计生、创建幸福家庭活动。广泛开展全民健身运动，加强面向群众的休闲健身场所建设，发展老年体育，培养竞技体育后备人才，建设市少体校新校区，争创国家级体育后备人才基地。推进统计制度方法改革，加强基层和部门统计工作。进一步做好民族宗教、档案、地方志、水文、防震和红十字会等工作。

　　促进社会和谐稳定。落实重大事项社会稳定风险评估办法，推进“三机制两体系一平台”建设，严密防范和坚决打击各类违法犯罪活动，完善治安视频监控和交通、消防安全设施，健全预防和处置医患纠纷“五位一体”长效机制。认真落实依法处理信访事项“路线图”，预防和妥善处置群体性事件。落实安全生产责任制，抓好安全隐患排查治理，有效遏制重特大安全事故发生。完善应急管理体制机制，加强应急队伍建设，提高处置突发公共事件的能力。加强城市社区建设，依法有序开展全市村（居）委会换届选举，树立管理服务典型。推进依法治市进程，抓好普法宣传教育和法律援助工作。加强民兵预备役建设，做好国防动员、人民防空工作，争创新一轮全国双拥模范城。

　　（五）着力加快生态工贸区建设，带动城乡一体化发展

　　抓紧实施生态工贸区规划。加快完善生态工贸区内城镇、园区详细规划和专业规划。依托4个重点园区，着力推进总投资698亿元的50个重点项目，组织策划和实施一批新的项目，促进更多大项目、好项目落地。探索生态工贸区管理运行机制，建立投融资、用地和人才保障机制。加大对接、沟通力度，力争省上尽快出台生态工贸区扶持政策，抓紧研究制定我市配套政策措施，推动生态工贸区加快发展。

　　推进中心城市建设。编制实施新一轮三明市城市总体规划，推动市区北扩南拓。北部新城建成徐碧新城（一期），加快徐碧村和列西龙岗旧城改造，推进贵溪洋新区建设，带动陈大、洋溪、富口等组团开发；南部新城加快推进台江、东霞片区和下洋中区建设，带动莘口、岩前、荆西等组团开发。抓好市县联办园区建设，壮大市区经济。加快三明市区、沙县和永安同城化工作，重点推进市区与沙县在公共服务、教育、医疗、通信等领域的同城化。加快中心城市快速通道建设，改造新市北路、南路和列东大桥，实施富兴路及南互通改造工程，加快停车场建设。提升城市管理水平，合理控制建筑密度和容积率，实施市区“一河两岸”城市景观改造，凸显绿地、水面和城市空间，力争市区建成区绿化覆盖率达42%以上。

　　提高县城规划建设水平。落实加快县域经济发展的政策措施，以特色产业为支撑，以县域城市为依托，推广泰宁和清流的城建模式，统筹抓好基础设施、建筑风格、城市色彩和公共服务，加快集聚工业、商贸、物流、旅游等产业，打造特色鲜明的块状经济，提升东南翼、西北翼的实力与发展水平。

　　（六）着力增总量壮实力，加快发展文化事业和文化产业

　　建设社会主义核心价值体系。深入开展中国特色社会主义理论体系学习教育，大力弘扬福建精神、三明精神和新时期老区苏区精神，深化拓展群众性精神文明创建活动，加快推进全国文明城市创建工作。加强未成年人思想道德建设，实施全民科学素质行动计划，不断提高人民群众的思想道德水平和科学文化素质。

　　大力发展文化事业。实施文化惠民工程，健全公共文化设施，加强县级数字影院建设，抓好农家书屋、公共电子阅览室、公益性上网场所建设，加快三明城市文化广场和市新图书馆、艺术馆、青少年宫建设，并向社会免费开放。加强非物质文化遗产的传承与保护，挖掘民族民间文化资源，争创国家级客家文化生态保护实验区。繁荣社会科学，发展文学艺术、广播电视、新闻出版事业。

　　加快发展文化产业。推进文化体制改革，健全文化市场综合执法运行机制。制定和落实扶持政策，培育10家重点文化企业，抓好43个重点文化产业项目，加快建设一批重点文化产业园区，推动文化旅游、文化创意、工艺美术等重点文化产业加快发展，增强文化产业实力。推进文化与科技、体育、信息、物流、建筑等产业融合发展，培育发展新兴文化业态，扩大文化产品和服务消费。

　　（七）着力创新体制机制，推动改革开放

　　推进重点领域改革。扎实抓好林权发证扫尾工作，加快建设永安全国林业改革与发展示范区，引导林农联营联合，促进林业规模化、集约化经营。稳定土地承包经营权，规范土地流转行为，全市所有乡镇基本建成土地流转服务平台。抓好政府机构改革评估，完成事业单位清理规范工作。加强税收征管，探索预算绩效管理，推进预算公开，严格控制“三公”等一般性财政支出。深化公共资源市场化配置改革。落实进城务工、经商人员落户政策。创新金融服务，支持发展创业投资，引进区外银行在我市设立分支机构、组建村镇银行，推动沙县农村金融改革试验区建设，全年新增贷款140亿元以上。做大担保业务总量，发展小额贷款公司，加快企业上市和发行债券步伐，推进交通、旅游、林业等企业集团筹建工作。

　　提升对外开放水平。聚全市之智，举全市之力，精心做好各项筹备工作，高水平办好世界客属第25届恳亲大会。创新招商机制，提高利用外资水平。落实鼓励外贸发展政策措施，抓好重点商品出口基地建设，扶持壮大20家出口龙头企业，提高纺织、林产等传统出口产品的档次，增加机电、高新技术等高附加值产品出口，鼓励扩大进口。加强与港澳地区、国外友城的沟通联系，做好海外华侨华人联络工作。拓展明台经贸、文化、旅游、科技、教育等领域的交流合作，加快三明台商投资区、清流台湾农民创业园、林博园建设，办好第八届海峡两岸林业博览会暨投资贸易洽谈会等重大活动。推进区域协作，对接闽东北、闽西南等经济协作区，加快省级山海协作示范园区建设。

　　壮大非公有制经济。进一步落实鼓励和扩大民间投资的政策，力争城镇以上民间投资达450亿元以上。扶持成长型非公有制企业发展，力争新增产值超亿元企业25家、税收超百万元企业50家。发展壮大商会经济，实施“回归工程”，加快发展总部经济。实施企业家素质提升工程，提高企业管理水平。

　　（八）着力加强生态建设，保护青山绿水蓝天

　　推进生态市建设。创建建宁、明溪、永安省级生态县（市），完成20个国家级、10个省级生态乡镇和200个市级以上生态村创建工作。严格生态功能区划管理，抓好自然保护区建设，推进生态公益林和生物多样性保护，强化水土保持和生态修复，加大矿区水土流失治理力度。强化水资源开发利用管理，严格河道采砂管理，加强饮用水源保护，推进市区应急水源和第二水源项目。实施“四绿”工程，完成造林绿化面积37万亩。强化耕地保护，保持耕地占补平衡，提高土地、矿产资源综合利用率。

　　强化节能减排。深化清洁生产试点城市工作，抓好28家省级以上循环经济试点企业（园区），开展清洁生产审核企业30家，完成重点节能改造项目30个、循环经济项目20个。加大淘汰落后产能力度，淘汰落后水泥产能120万吨、造纸产能20万吨。全面落实环境保护“一岗双责”，严格控制污染物排放总量。着力实施51个重点减排项目，开展重点行业、工业园区环境污染整治，加强重金属污染治理，实现垃圾和污水处理设施达标运行，推进重点流域水环境综合整治，深化畜禽养殖污染治理。强化市区大气环境综合整治，加大市区粉尘治理力度，加快淘汰老旧机动车，力争市区空气优良天数占总天数的90%以上。

　　四、加强政府自身建设

　　推动三明科学发展跨越发展，加快建设开放三明幸福三明，对政府自身建设提出更高要求。各级政府要勇于担当使命，依靠各界力量，建设全心全意为人民的服务型政府。

　　（一）进一步解放思想，激发创新活力。立足本职、从我做起，用科学先进的理念实践先行先试的要求。创新工作机制，严格落实制度，配强项目团队，增强运作能力，破解发展难题。强化激励措施，严格兑现奖惩，调动方方面面抓发展的积极性。不求所有、但求所在，努力吸引更多企业落户三明、支撑发展，营造全社会干事创业的良好氛围。

　　（二）进一步改进作风，提高服务效能。坚持施政为民，深入开展“下基层、进企业、解难题”活动，尽职尽责服务，让企业和群众少跑路、不受累。加强政府绩效管理，加大治庸治懒治散力度，严格执行效能问责。弘扬求真务实作风，多办实事好事，致力于打基础、添后劲、利长远。加大简政放权力度，能减则减、能放则放、能优则优。推进行政审批制度改革，加强行政服务中心建设，力争市县各类项目审批时限在法定时间内再压缩50%以上，促进行政审批提速。

　　（三）进一步加强法制，推进依法行政。认真对人大及其常委会负责并报告工作，依法接受人大及其常委会法律监督和工作监督，自觉接受政协民主监督，办好人大代表建议和政协提案。加强与各民主党派、工商联、无党派人士的联系与协商，充分发挥工会、共青团、妇联等人民团体的作用。健全科学决策、民主决策、依法决策机制，深化政务公开和政府信息公开，做到规范执法、公正执法、文明执法。加强行政复议工作，有效化解行政争议。

　　（四）进一步从严治政，树立良好风气。认真落实党风廉政建设责任制，自觉遵守廉政准则，深入开展反腐倡廉教育，筑牢拒腐防变的思想道德防线。构建惩治和预防腐败体系，规范土地和矿产资源开发、公共资源交易、重大工程建设、政府采购等管理，从源头上预防腐败。强化行政监察和审计监督，严格财政收支监督，严肃查处违纪违法行为，坚决纠正损害群众利益的不正之风，以反腐倡廉的实际成效取信于民。各位代表！站在新的发展起点，我们倍感责任重大。让我们更加紧密地团结在以胡锦涛同志为总书记的党中央周围，高举中国特色社会主义伟大旗帜，以邓小平理论和“三个代表”重要思想为指导，深入贯彻落实科学发展观，在市委的正确领导下，团结和依靠全市人民，同心协力，开拓进取，为加快建设开放三明幸福三明而努力奋斗，以优异成绩迎接党的十八大胜利召开！
